# Assessment of long-term large deformation in deep roadways due to roof fracturing impact loading

**DOI:** 10.1038/s41598-023-30792-9

**Published:** 2023-03-08

**Authors:** Wanpeng Huang, Naser Golsanami, Chengguo Zhang, Ismet Canbulat, Guangming Xin, Gang Sun, Lishuai Jiang

**Affiliations:** 1 College of Energy and Mining Engineering, Shandong University of Science and Technology, Qingdao, 266590 China; 2 School of Minerals and Energy Resources Engineering, University of New South Wales, Sydney, NSW 2052 Australia; 3 State Key Laboratory of Mining Disaster Prevention and Control, Shandong University of Science and Technology, Qingdao, 266590 China; 4 Yangcheng Coal Mine, Jining Energy Development Group Co. Ltd., Jining, 272000 China

**Keywords:** Geodynamics, Structural geology

## Abstract

The rock mass around deep roadways has obvious creep characteristics in high-stress environments. Meanwhile, the cyclic impact load induced by roof fracturing also causes dynamic damage to the surrounding rock, leading to long-term large deformation. This paper examined the rock mass deformation mechanism around deep roadways based on the theory of rock creep perturbation effect considering perturbation sensitive zone. This study proposed a long-term stability control guideline for deep roadways under dynamic load. An innovative support system was developed for deep roadways, with concrete-filled steel tubular support being recommended as the main supporting body. A case study was conducted to validate the proposed supporting system. Monitoring over one year in the case study mine showed that the overall convergence deformation of the roadway was 35 mm, indicating that the roadway’s long-term large deformation induced by creep perturbation was effectively controlled by using the proposed bearing circle support system.

## Introduction

As mining goes deeper, the stress concentration caused by roadway excavation increases significantly, and the rock mass shows creep characteristics. The deformation of the roadway has pronounced time effect characteristics. The short-term equilibrium state of the rock mass in creep state could be easily disturbed due to the impact of the external perturbation load. This increases the creep deformation rate of the roadway, and could even lead to dynamic disasters^1–5^. In deep mining operations, the dynamic impact load is mainly caused by the sudden fracturing of the competent main roof units. This impact load is termed as roof-fractured impacting loading or RFIL in this paper. This type of impact load does not cause the instantaneousdamage of a roadway’s surrounding rock. However, it will cause a certain perturbation deformation of the surrounding rock. When this type of deformation is superimposed with the creep deformation of the surrounding rock, the deformation rate of the roadway increases, resulting in long-term large deformation. At the same time, the RFIL has the characteristics of cyclic impacts. With the periodic fracturing of the main roof above the face, the impact load will act on the surrounding rock repeatedly, exacerbating the long-term large deformation of the deep roadway. Rock burst in deep mining has been increasingly investigated globally. The mechanisms of rock burst, monitoring and early warning of impacting load, and pressure relief technology during mining have been thoroughly studied, with many innovative results achieved^6–10^. Nevertheless, most of the existing research work has focused on the dynamic disasters of a deep roadway under the influence of impact loads with high energy levels (impact energy > 10^5^ J). However, the energy level induced by roof strata fracture in deep mining is much lower than that of a rock burst, being approximately 10^3^–10^5^ J. The long-term large deformation mechanism and quantitative support design of the surrounding rock of deep roadways under RFIL has not been investigated thoroughly, with a limited number of relevant studies.

Therefore, this study conducted theoretical assessment of the support system and verified them using a case study in a coal mine in China. The theory of the perturbation effect of creeping rock was tested, where the influence mechanism of RFIL on the surrounding rock in the creep state was analysed. Then, a quantitative design method for the support system of the deep roadway affected by the dynamic pressure was proposed.

## Long-term deformation mechanism of deep roadways affected by RFIL

### Perturbation effect of creeping rock under RFIL

The perturbation effect of creeping rock describes the mechanical phenomenon that the rock’s creep deformation will increase at a certain stress level when affected by external impact loads (such as shooting vibration etc.). It is a critical research area for the prevention and control of dynamic disasters in deep roadways impacted by the external dynamic load. However, in the existing literature of rock dynamics, the research focus on this issue is still limited. The authors’ research group previously proposed the concept of the perturbation effect of creeping rock^11,12^ and developed a new type of equipment to test the perturbation effect of creeping rock. Based on this new testing system, the process and method of the dynamic impacting experiment of creeping rock were introduced at a laboratory scale. In addition, some preliminary research on typical creeping sandstone specimens under RFIL was conducted, considering the constitutive relationship of the rock’s perturbation deformation.

**Figure 1:**
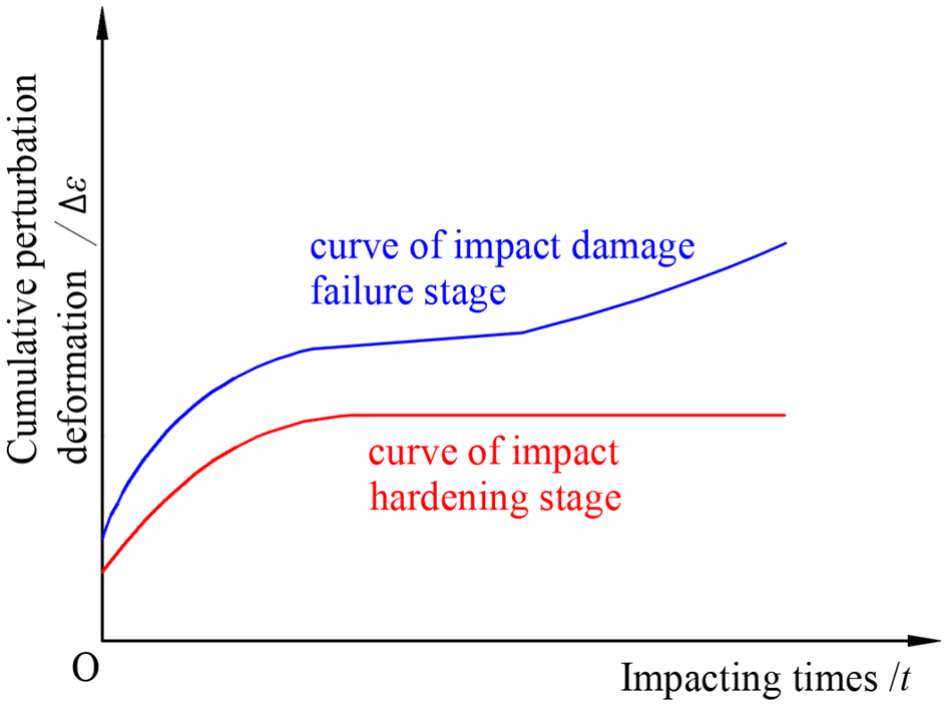
Curve of the rock’s cumulative perturbation deformation.

The impact load acts on the creeping rock in the form of shock waves, causing the rock to generate the corresponding disturbing deformation increment. Under varying static stress conditions, rocks typically exhibit two different responses to external impact loads. At a lower static stress level, the rock is in the initial stage of creep, showing an obvious hardening characteristic under the effect of external cyclic impact load. The curve of the rock’s cumulative perturbation deformation at this stage is shown in Fig. 1. The axial cumulative perturbation deformation of the rock increases rapidly during the first few impacts, then the growth rate gradually decreases to zero. This implies that the subsequent impact gradually fails to cause a further increase in the deformation, and the rock shows impact hardening characteristics. It indicates that the rock is not sensitive to the impact of external perturbation load at this stage, and the impact has less dynamic damage on the creeping rock in this stage. At a higher static stress level, after the rock enters the stable creep stage, it begins to show the damage and destruction characteristics under the action of external cyclic impact load, as shown in Fig. 1. The axial cumulative perturbation deformation of the rock no longer shows obvious signs of attenuation with increasing impacts, but a development trend in the form of stepwise growth. The damage within the rock begins to intensify, and the rock deformation moves from the impact hardening stage to the damage failure stage^13^. This shows that the rock has already begun to become sensitive to the external impact load, and the impact has more damage on the creeping rock in this stage. The cumulative perturbation deformation and creeping deformation of the rock will cause long-term large deformation until failure. At the same time, as the intensity of the impact load increases, the curve of the rock’s cumulative perturbation deformation is transformed from the attenuation stage to the continuous development stage in advance. As a result, the rock begins to become more prone to external perturbation at lower static stress levels.

**Figure 2:**
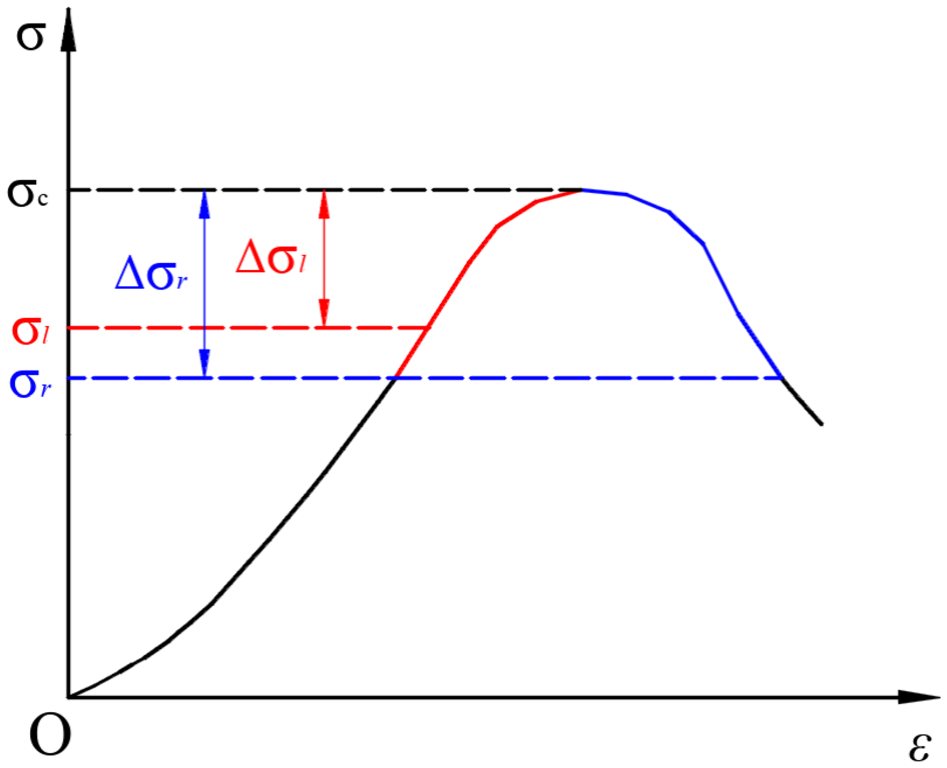
Curve of the rock’s “limit strength neighborhood”.

Based on different sensitivities of the rock to the external impacts under various static stress conditions, the authors propose the new mechanical concepts of “limit strength neighbourhood” and “anti-perturbation strength” of the rock. The limit strength neighbourhood of rock refers to the stress state of the rock. That is, under a simple compressive (tensile, shear) state, the rock has a limit strength $\sigma_{c}$; then, given a value $\Delta \sigma$ based on a certain condition, if the static stress applied on the rock satisfies the following formula, it is in the neighbourhood of the rock’s limit strength^14^:

1 $$ \left| {\sigma_{c} - \sigma } \right| \le \Delta \sigma $$

where $\Delta \sigma$ is the width range of the limit strength neighbourhood of the rock as indicated in Fig. 2. The rock’s limit strength neighbourhood can be further subdivided into the left and right neighbourhood. If the static stress acting on the rock has not yet reached the peak strength, it is said to be within the left neighbourhood. The left neighbourhood’s width can be represented by $\Delta \sigma_{l}$, $\Delta \sigma_{l} = \sigma_{c} - \sigma_{l}$, where $\sigma_{l}$ is the strength threshold of the rock’s left neighbourhood of the limit strength. If the static stress exceedes the peak strength of the rock, it is within the right neighbourhood. The right neighbourhood’s width can be represented by $\Delta \sigma_{r}$. When the applied stress is in two different neighbourhoods, the perturbation deformation characteristics of the rock are different. Previous studies have focused on the perturbation deformation characteristics of the rock when the static stress is in the left neighbourhood. Limited research has been conducted to characterise the situation where the static stress is in the right neighbourhood.

There are different mechanical behavior of the rock when the applied static stress is in the neighborhood of the limit strength or outside the neighborhood. If the static stress is about the limit strength, the rock is more sensitive to external impact perturbation and its deformation is in the stage of damage and destruction. In contrast, when the static stress is outside the neighbourhood, the rock is not sensitive to external impact perturbation, and the impact will not cause perturbation deformation or the resultant deformation will gradually decay. Under such a condition, the rock’s deformation will occur in the impact hardening stage. According to Fig. 2, the strength threshold $\sigma_{l}$ of the rock’s left neighbourhood is the critical index of the non-sensitive area and sensitive area of the rock against the impact disturbance. Therefore, $\sigma_{l}$ could be defined as the rock’s anti-perturbation strength. This parameter is an important indicator for assessing the effect on the rock of an external perturbation load. For deep rock engineering, by comparing the relationship between the static stress level applied on the rock and its anti-disturbance strength, we can estimate whether the surrounding rock is currently sensitive or non-sensitive to the external perturbation load. This provides a basis for further analysis of the impact deformation mechanism of the surrounding rock and appropriate support design. The parameter of a rock’s anti-perturbation strength can be obtained from the testing method of rock creep perturbation experiment^12^. According to the results, for medium to hard sandstone (siltstone and red sandstone), the anti-perturbation strength index is basically equivalent to the sandstone’s long-term strength, which is $\sigma_{l} = \sigma_{s}$. This reveals an important understanding that the rock starts to become sensitive to external impact perturbation when it enters the stable creep stage.

### Long-term deformation mechanism of creeping rock affected by the impact loading

The rock mass in deep roadways is affected by both high static stress and cyclic external impact load, and the characteristics of its deformation are long term and dynamically gradual. This is much more complicated than the surrounding rock deformation in roadways on low stress environment. Therefore, the deformation theory of shallow roadways appears to be not suitable for analysing the long-term large deformation mechanism of deep roadways. The present study adopted the basic theory of rock creep perturbation effect to characterise the mechanism of deformation of deep roadways by redefining the surrounding rock’s state zone and analysing the evolution law of the dynamic stress field.

#### Reclassification model of the surrounding rock’s state zone

**Figure 3:**
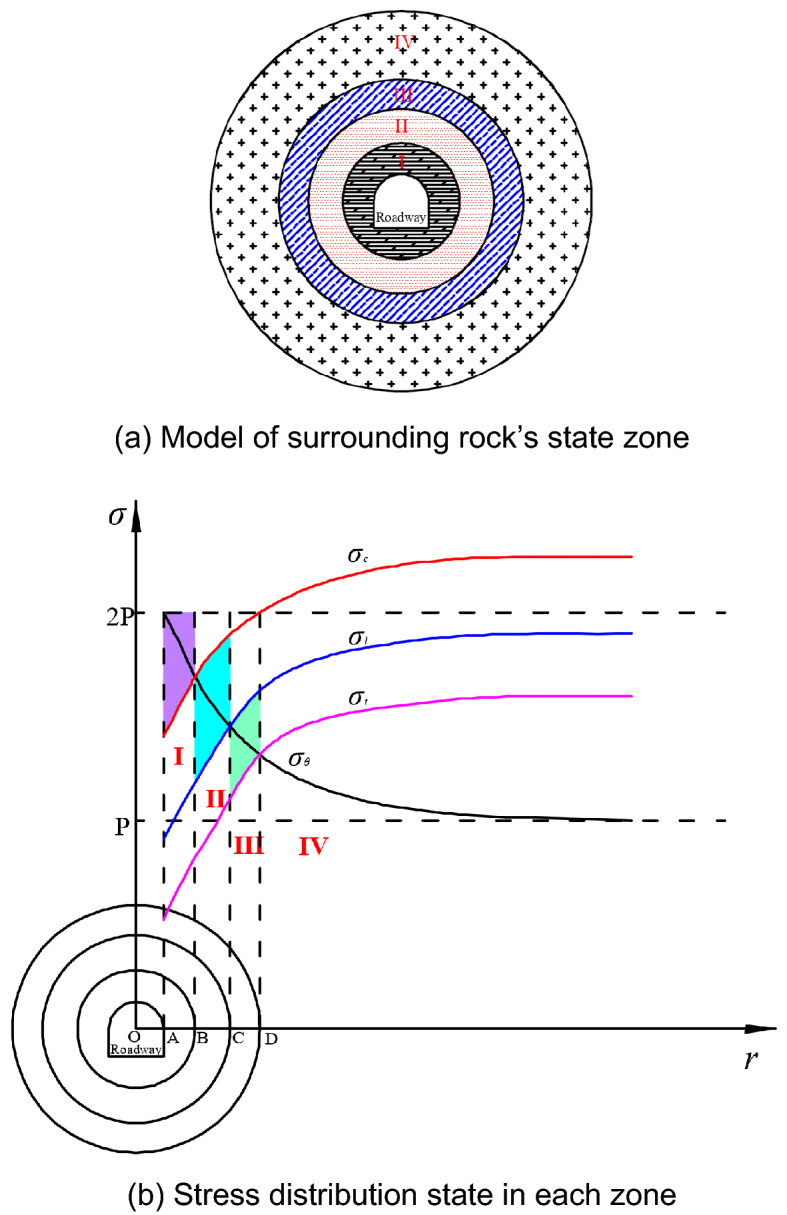
Classification model and stress distribution of surrounding rock’s state zone under creep perturbation (where I: Fractured zone; II: Perturbation sensitive zone (stable creeping zone); III: Perturbation insensitive zone (attenuated creeping zone); IV: Elastic zone; *P*: Original stress level; $\sigma_{\theta }$:Tangential concentrated stress on surrounding rock; $\sigma_{c}$: Ultimate strength of surrounding rock; $\sigma_{l}$: Anti-perturbation strength of surrounding rock; $\sigma_{t}$: Threshold strength of surrounding rock when creep occurs).

In the traditional theory of surrounding rock deformation in shallow roadways, the rock’s state zone is divided into three zones: fractured zone, plastic zone, and elastic zone after roadway excavation. This classification is based on the static stress level and deformation characteristics of the surrounding rock. When considering the creep perturbation characteristics of the rock and comprehensively analysing it with the high static stress level and dynamic impact load, it is understood that the surrounding rock’s state zone will change after roadway excavation. Then a new classification model for the surrounding rock’s state zone was established^15–17^. This model is illustrated in Fig. 3a. The model divides the surrounding rock’s state zone after roadway excavation into four different zones: fractured zone, perturbation sensitive zone, perturbation insensitive zone, and elastic zone. The contrasting relationship between the static stress level and the surrounding rock’s mechanical strength in each zone and its developing law are shown in Fig. 3b.

After excavation of a deep roadway, a larger stress concentration will occur within a certain range of the surrounding rock where the maximum concentrated tangential stress at the roadway rib can reach about twice the original stress level. Going further into the surrounding rock, the concentrated stress will gradually attenuate and eventually decrease to the original stress level. This stress curve is shown by the black curve in Fig. 3b. With the excavation of the roadway, the surrounding rock mass at the rib changes from the three-way compression state to the one-way compression state, and its strength decreases rapidly. On the other hand, the lateral confining pressure that the rock mass increases when going inside the surrounding rock. The corresponding strength of the surrounding rock continues to increase and eventually reaches a stable state. In Fig. 3b, the red, blue, and pink curves represent the developing rule of the ultimate strength, anti-perturbation strength, and creep threshold strength of the surrounding rock from the roadway rib into the pillar, respectively,. According to the relationship between the static concentrated stress on the surrounding rock and the different strengths of the engineering rock mass, four zones can be suggested: the fractured zone, perturbation sensitive zone, perturbation insensitive zone, and elastic zone. The stress state and deformation characteristics of the surrounding rock in each zone are different and are summarised as follows:

I Fractured zone: In this zone, the large concentrated static stress exceeds the compressive ultimate strength of the rock mass. The stress relationship is $\sigma_{\theta } \ge \sigma_{c}$ (shown in the purple area in Fig. 3b). As a result, a large number of internal cracks in the surrounding rock initiates, and the strength is reduced to the residual strength. After roadway excavation, the damage will occur in this zone within a short time, and the surrounding rock will lose its original bearing capacity.
II Perturbation sensitive zone: After the surrounding rock of the roadway is failed, the concentration of the static stress decreases. At the same time, the rock surrounding the fractured zone can provide a small lateral confining pressure to the internal surrounding rocks, making them enter a three-way compression state. This increases the various strengths of the inner surrounding rock. In the perturbation-sensitive zone, the reduced concentrated static stress is higher than the rock’s anti-perturbation strength but lower than its ultimate strength. The stress relationship is $\sigma_{l} \le \sigma_{\theta } \le \sigma_{c}$ (as shown in the blue area in Fig. 3b). Therefore, the surrounding rock in this zone will not break in the short term, but it is still in an unstable state and is sensitive to external perturbations.
III Perturbation insensitive zone: The stress relationship in this zone is $\sigma_{t} \le \sigma_{\theta } \le \sigma_{l}$ (the green area in Fig. 3b). Following the evolution law of the stress field described in the section below, the further reduced concentrated static stress is higher than the creep strength threshold but lower than the anti-perturbation strength of the surrounding rock. Therefore, the surrounding rock in this zone is not sensitive to external impact perturbations, showing the characteristics of impact hardening. The deformation is mainly the decayed creep deformation, but does not cause long-term damage to the surrounding rock.
IV Elastic zone: The surrounding rock in this zone is basically in the state of elastic deformation, which has little effect on the roadway deformation.

#### Evolution law of the stress field and long-term deformation mechanism

**Figure 4:**
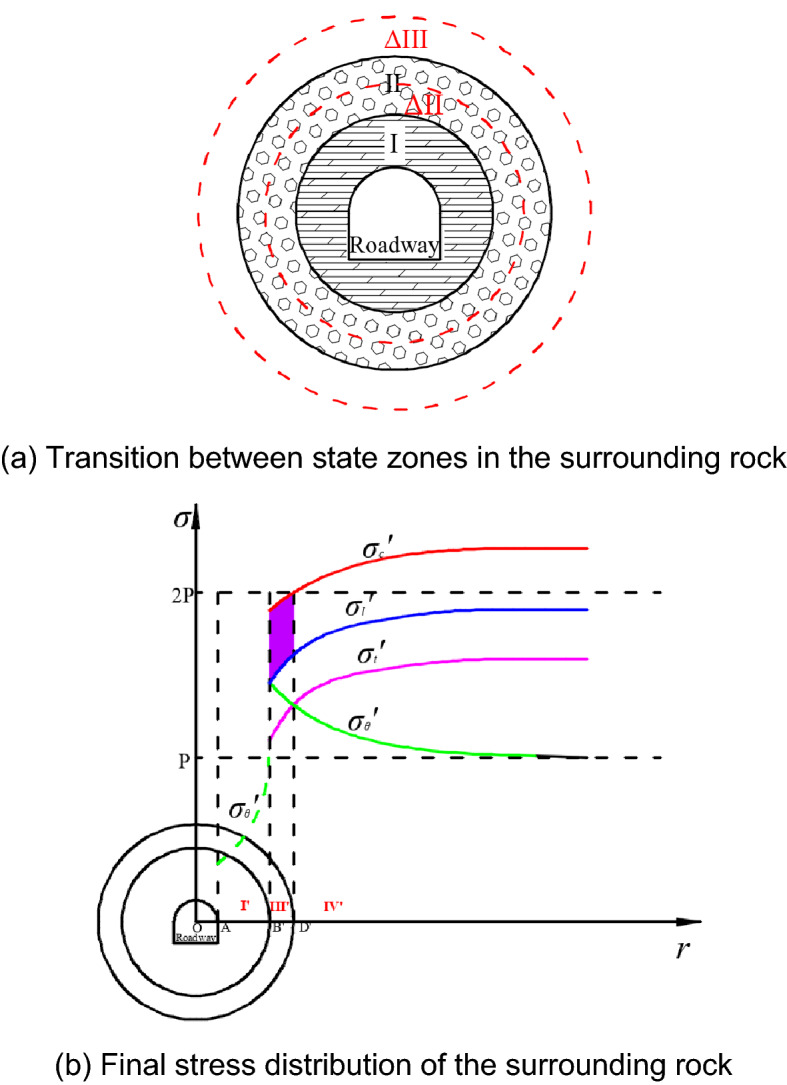
Evolution law of the stress field in the surrounding rock in the creep perturbation state.

After excavation of a deep roadway, the major deformation of the roadway are induced from the dilitant deformation and the integral extrusion. As for the rock mass in the perturbation-sensitive zone, creep deformation occurs as a result of the external dynamic loading, then the zone will extend to a fractured zone, which is gradually increasing as time. Because of the presence of the perturbation-sensitive zone, an irreversible cumulative perturbation deformation of the surrounding rock in zone II will occur after the rock is impacted by the RFIL. When the deformation continues to increase to the ultimate deformation of the surrounding rock, this zone will transform into the fractured zone, and the perturbation-sensitive zone will continue to move into the deeper surrounding rock. This dynamic process is represented in Fig. 4a. After the action of the impact load, part $\Delta$ II that originally belonged to zone II evolves into the fractured zone, increasing the range of zone I. The perturbation-sensitive zone continues to shift into the deeper surrounding rock, making the part $\Delta$ III that originally belonged to zone III evolve into the perturbation-sensitive zone.

During the dynamic evolution of the perturbation-sensitive zone into the deeper surrounding rock, the concentrated static stress is further reduced, and the ultimate strength and anti-perturbation strength of the internal surrounding rock continue to increase accordingly. When the decreasing concentrated static stress peak is equal to the increasing anti-perturbation strength of the surrounding rock, the surrounding rock reaches a relatively balanced state. The distribution of stress in this process is shown in Fig. 4b. In this balanced state, the perturbation-sensitive zone of the surrounding rock eventually disappears, and the fractured zone develops to the maximum. In the final fractured zone I', the concentrated static stress reduces to a lower level; and the surrounding rock only has residual strength. After the perturbation-sensitive zone disappears, the outside of the fractured zone is directly the perturbation insensitive zone III'. In this zone, the anti-perturbation strength of the surrounding rock is greater than the concentrated static stress, which is $\sigma_{\theta } \le \sigma_{l}$ (the purple area in Fig. 4b). The concentrated static stress is outside the limited strength neighbourhood of the surrounding rock, so the engineering rock is no longer affected by the external impact load.

In summary, the long-term large deformation and instability mechanism of a deep roadway under the action of RFIL can be analysed and obtained. This deformation and instability are due to the continuous transfer of the perturbation-sensitive zone into the interior surrounding rock under the double effect of greater static stress and the external impact load. If the dynamic developing progress of the perturbation sensitive zone cannot be controlled, a large extent of fractured zone will eventually form, resulting in the long-term large deformation and failure of the deep roadway. This deformation mechanism based on the theory of rock creep perturbation effect can better explain the long-term large deformation and failure phenomenon of deep roadways under dynamic load.

## Control principle and support design of deep roadways affected by RFIL

After excavation of a deep roadway, the surrounding rock will soon be damaged under the double action of dynamic and static loads if there is no artificial support^18,19^. According to the above analysis of the dynamic stress field evolution and the deep roadway deformation mechanism, to ensure the long-term stability of a deep roadway, there are two key aspects for the ground support design. The first one is to control the short-term bulking deformation of the surrounding rock in the fractured zone, and then to control the dynamic transfer and evolution of the perturbation-sensitive zone into the interior surrounding rock. This study proposes an innovative supporting principle for deep roadways affected by RFIL, named the “bearing circle”, together with our support elements as a system.

### The “bearing circle” supporting principle

**Figure 5:**
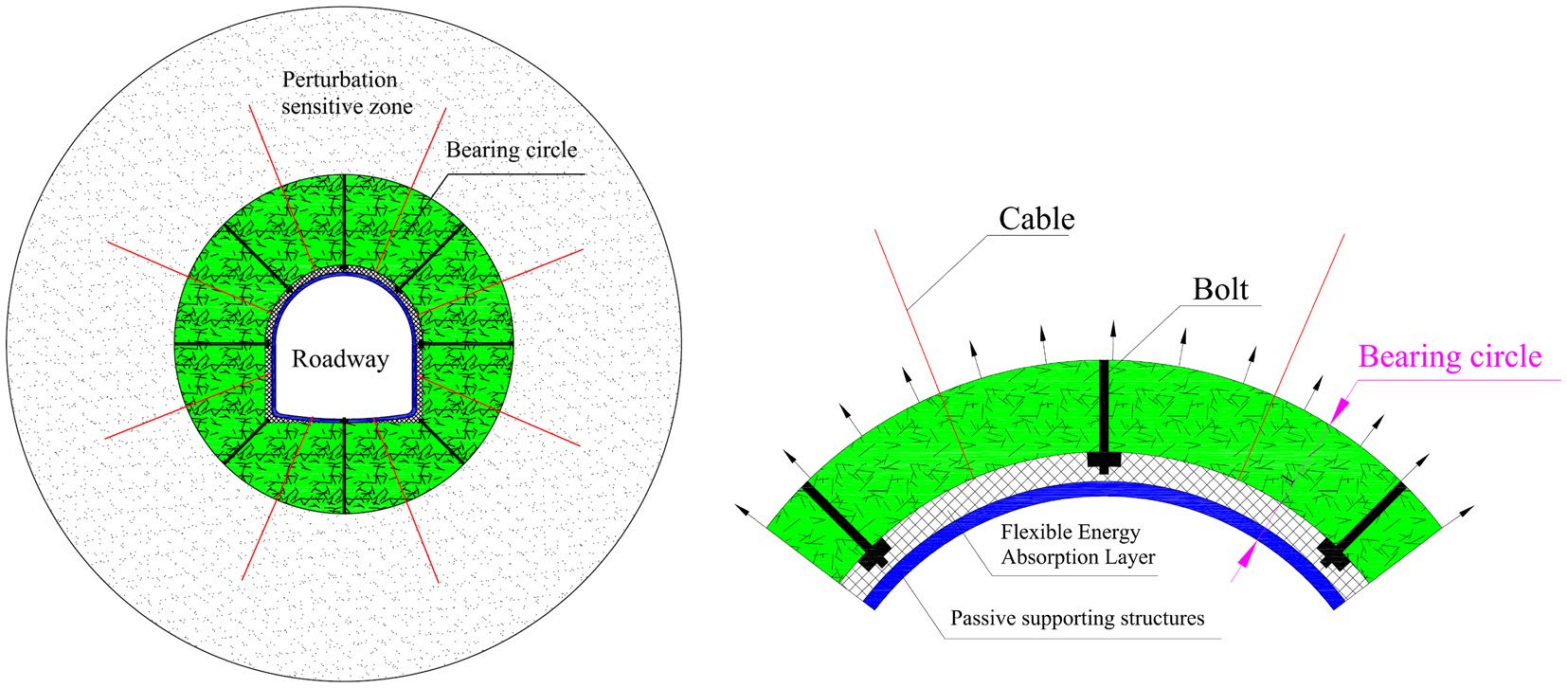
Structure of the surrounding rock “bearing circle”.

The main principle of the “bearing circle” anti-impact supporting technology is to construct a circle within the fractured zone around the roadway which is reinforced by supporting structures, as shown in the green area in Fig. 5. The circle has two parts: the artificially passive supporting structures within the roadway and in the surrounding rock in the anchorage zone.

The circle functions to strengthen the integrity and bearing capacity of the surrounding rock in the fractured zone, control the short-term bulking large deformation of the fractured zone, and improve the stability and impact resistance of the surrounding rock in the anchorage zone. The circle can also provide a higher lateral confining pressure to the surrounding rock in the perturbation sensitive zone, increasing the anti-perturbation strength and ultimate strength of the surrounding rock in the entire perturbation sensitive zone. The bearing circle can also artificially redistribute the high concentrated static stress in this zone from the limit strength neighborhood range of the surrounding rock, and improve the bearing capacity and impact resistance of the surrounding rock in the zone. The greater the lateral confining force provided by the “bearing circle”, the earlier the high static stress in the perturbation sensitive zone exits the rock limit strength neighbourhood range, and the better the supporting effect will be^20–22^.

**Figure 6:**
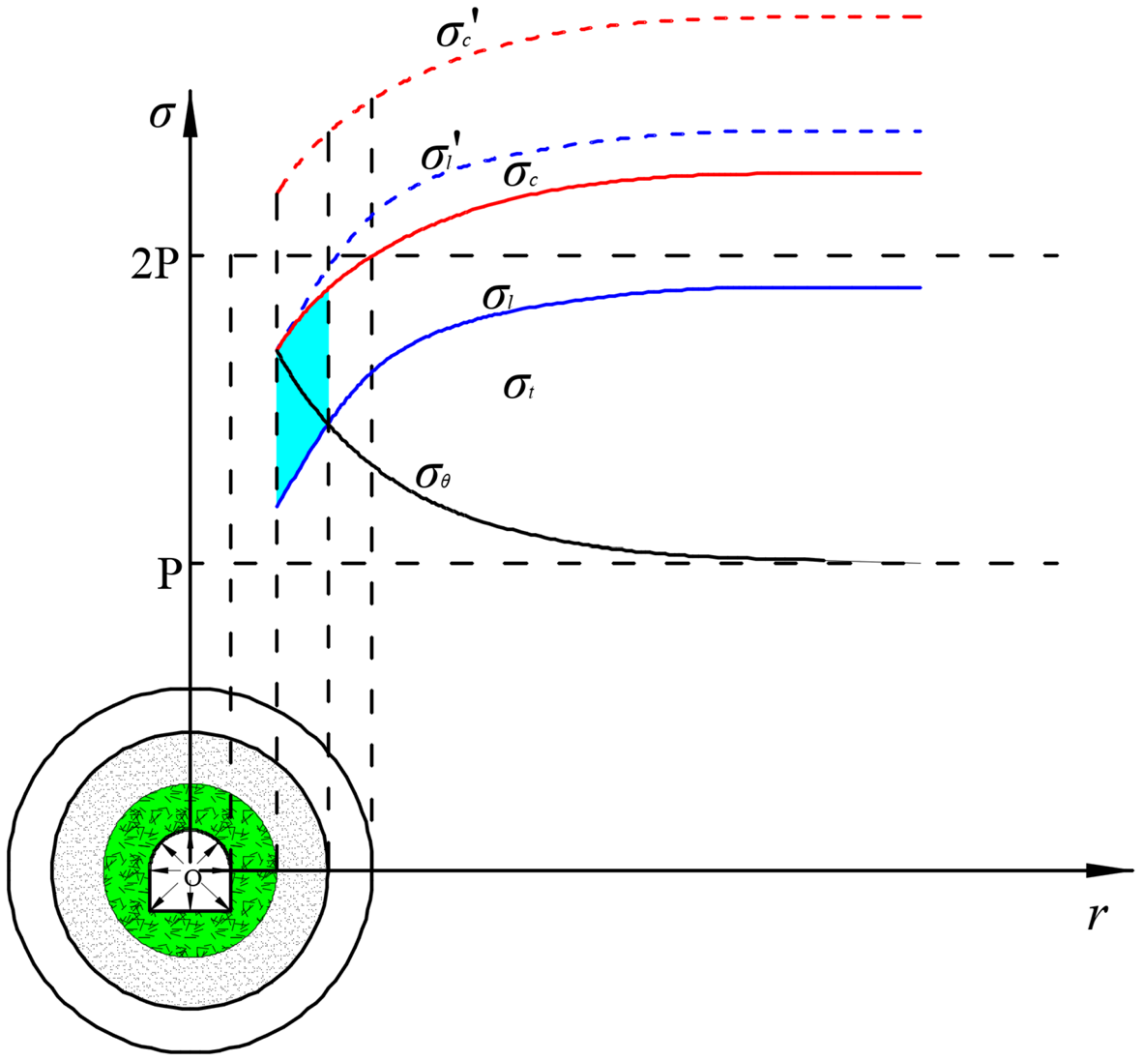
Stress distribution state of the surrounding rock after the “bearing circle” is established.

Figure 6 shows the stress distribution state of the surrounding rock after the “bearing circle” is implemented. In this figure, due to the increase of the surrounding rock’s strength in the perturbation-sensitive zone under the support of the “bearing circle”, the curves of the anti-perturbation strength and ultimate strength both shift upwards. When the increased anti-perturbation strength is higher than the peak value of the concentrated static stress in this zone, this part of the surrounding rock will be transformed into the perturbation insensitive zone, and the perturbation sensitive zone disappears. At this time, the external impact load no longer disturbs the surrounding rock of the roadway. Through the mechanical effects of these two aspects, the “bearing circle” surrounding the rock structure can effectively control the short-term bulking deformation and long-term perturbation deformation of the deep roadway, ensuring the long-term stability of the deep roadway.

### Composition of the “bearing circle” supporting technology

The “bearing circle” support system can resist high pressure and external impact. The mechanical properties of this structure are determined by the specific properties of the support capacity of the elements used. The “bearing circle” structure proposed in this paper uses the following four types of support elements.

1. Bolt, mesh and concrete spray layer

The supporting effect of the bolt significantly improves the cohesion and internal friction angle of the fractured surrounding rock, thereby increasing the strength of the surrounding rock in the anchorage zone. At the same time, the bolt can improve the integrity of the anchorage zone and strengthen its bearing capacity. The main function of the metal mesh and the concrete spraying layer is to provide surface support and coverage. At the same time, these two structures can spread the force of the bolts evenly to the surrounding rock and establish a stable anchorage zone around the roadway.

(2) Cable bolts support

As for cable bolt, one end of the cable is anchored in the deep interior of the surrounding rock, and the other end is closely attached to the roadway wall through the locking device. This suspends the anchorage zone in the more stable interior strata. It can also increase the friction between the “bearing circle” and the perturbation-sensitive zone, and prevent the layer sliding between the surrounding strata.

(3) Concrete-filled steel tubular support (CSTS)

The CSTS is a new type of high-strength structure to support the soft surrounding rock of deep roadways. The structural composition, mechanical properties and engineering application of CSTS were previously introduced^21^. The ultimate bearing capacity of the CSTS is about 3–5 times that of the same type U-type steel support. Therefore, installing it in the roadway as a passive supporting structure can provide extra radial force for the “bearing circle”. This is equivalent to applying a large amount of lateral confining pressure to the surrounding rock improving its bearing capacity. The force provided by the CSTS can also act on the surrounding rock in the perturbation-sensitive zone through the “bearing circle”, providing a substantial increase in its anti-perturbation strength and ultimate strength. Of note is that the filling material between rock surface and concrete-filled steel tubular support is an important link to achieve energy absorption capacity. It is usually filled with yielding material, such as timber plates, to allow further deformation prior to the close contact between the rock and the concrete-filled steel tubular support.

(4) Surrounding rock grouting

During the development of the surrounding rock’s deformation, the internal cracks in the rock mass will gradually develop as the number of deformation increases. When the cracks develop to a certain extent, they will cause the destruction and instability of the surrounding rock. Surrounding rock grouting technology can improve the physical and mechanical properties of the fractured rock mass from the inside the surrounding rock, increase the static and dynamic elastic modulus of the rock mass, weaken the anisotropy of the rock mass, ensure the complete unity of the surrounding rock and improve its bearing capacity. At the same time, the internal cracks are filled by grouting into the surrounding rock, which can improve the cohesion *C* and internal friction angle of the rock mass and therefore increase the strength of the fractured rock.

### Mechanical analysis and determination of support capacity

1. Mechanical analysis in perturbation-sensitive zone

According to the analysis, the condition for the long-term stability of a roadway is to redistribute the concentrated static stress in the perturbation-sensitive zone from the limit strength neighbourhood of the surrounding rock. The mechanical condition that should be satisfied is $\left| {\sigma_{c} - \sigma_{\theta } } \right| \ge \Delta \sigma$. In the entire perturbation-sensitive zone, the concentrated static stress peak at the outer boundary (i.e., point B in Fig. 3) is the highest, while the anti-perturbation strength of the surrounding rock is the lowest. Therefore, the above mechanical condition can be simplified to the mechanical judging condition at the boundary of the region, which is:

2 $$ \sigma_{\theta B} \le \sigma_{lB}^{\prime } $$

where $\sigma_{\theta B}$ is the concentrated static stress peak at the outer boundary of the perturbation sensitive zone, that is, the concentrated static stress applied on the surrounding rock at point B in Fig. 3 and $\sigma_{lB}^{\prime }$ is the increased anti-perturbation strength of the surrounding rock at point B after being supported.

After roadway excavation, the fractured zone will form in a short time, and the peak of concentrated static stress will immediately be transferred to the boundary of the fractured zone and perturbation-sensitive zone. According to rock mechanics theory, the peak value can be calculated according to the following formula:

3 $$ \sigma_{\theta B} = P(1 + \sin \varphi ) + C\cos \varphi $$

where *P* is the original stress level; *C* is the cohesion of surrounding rock in the original state; and $\varphi$ is the internal friction angle of surrounding rock in the original state.

The surrounding rock strength at the outer boundary of the perturbation sensitive zone can be considered as the uniaxial compressive strength assuming that the surrounding rock in the fractured zone completely loses its bearing capacity and its lateral support for the perturbation sensitive zone. This kind of safety consideration is allowed in engineering design. At the same time, because the formation of the fractured zone occurs in a relatively short period of time, it can be assumed that the surrounding rock in the perturbation-sensitive zone during this stage still follows the Coulomb–Mohr strength criterion^23,24^. After the artificial support structure is implemented, the “bearing circle” is formed. Assuming that the lateral support strength provided by the circle is $\sigma_{SB}$, then the anti-perturbation strength $\sigma_{lB}^{\prime }$ of the surrounding rock at the outer boundary of the perturbation-sensitive zone can be calculated by the following formula:

4 $$ \sigma_{lB}^{\prime } = \frac{1 + \sin \varphi }{{1 - \sin \varphi }}\sigma_{SB} + \sigma_{lB} $$

where $\sigma_{lB}$ is the anti-perturbation strength of surrounding rock in the original state at point B under uniaxial compression. This parameter can be tested by the rock creep perturbation experiment as mentioned previously.

(2) Mechanical analysis in the “bearing circle” zone

In Eq. (4), to get the parameter $\sigma_{lB}^{\prime }$, the key is to determine the parameter $\sigma_{SB}$, which is the lateral support strength provided by the “bearing circle” structure. As noted in Section "Composition of the “bearing circle” supporting technology", the “bearing circle” structure uses several supported elements: a bolt, metal mesh and concrete spray layer, cables, and CSTS. Assuming that (i) the supporting strength provided by the suspension effect of the cable on the wall of the roadway is $\sigma_{{_{S} }}^{1}$, (ii) the supporting strength provided by the CSTS is $\sigma_{{_{S} }}^{2}$, (iii) the original fractured zone is transformed into the anchorage zone after being supported by bolts, and (iv) the surrounding rock in the anchorage zone still meets the Coulomb–Mohr strength criterion after being anchored, we can conclude that the static balance equation in the anchorage zone can be written as:

5 $$ \sigma_{s} + r\frac{{d\sigma_{s} }}{dr} - \frac{{1 + \sin \varphi^{\prime}}}{{1 - \sin \varphi^{\prime}}}\sigma_{s} - \frac{{2C^{\prime}\cos \varphi^{\prime}}}{{1 - \sin \varphi^{\prime}}} = 0 $$

where $\sigma_{s}$ is the radial stress at any position in the anchorage zone, *r* is the radius at any position in the anchorage zone, and $\varphi^{\prime}$ and $C^{\prime}$ are the increased internal friction angle and cohesion of the surrounding rock strengthened in the anchorage zone, respectively.

Solving the differential Eq. (5) gives: $\sigma_{S} = Ar^{2\sin \varphi ^{\prime}/(1 - \sin \varphi ^{\prime})} - C^{\prime}\cot \varphi ^{\prime}$, where A is the integral constant. According to the inner boundary conditions of the anchorage zone, when $r = R_{0}$ (the radius of the roadway), $\sigma_{S} = \sigma_{s}^{1} + \sigma_{s}^{2}$. So *A* can be calculated as $A = (\sigma_{s}^{1} + \sigma_{s}^{2} + C^{\prime}\cot \varphi^{\prime})/R_{0}^{{^{2\sin \varphi ^{\prime}/(1 - \sin \varphi ^{\prime})} }}$. By substituting this into Eq. (5), the formula for $\sigma_{s}$ can be obtained as:

6 $$ \sigma_{s} = (\sigma_{s}^{1} + \sigma_{s}^{2} + C^{\prime}\cot \varphi^{\prime})\left( {r/R_{0} } \right)^{{\frac{{2\sin \varphi^{\prime}}}{{1 - \sin \varphi^{\prime}}}}} - C^{\prime}\cot \varphi^{\prime} $$

The radius of the anchorage zone is the effective length of the bolt *R*_1_; therefore, the radial stress of the surrounding rock at the outer boundary of the anchorage zone at *r* = *R*_1_, i.e., the lateral support strength provided by the “bearing circle” to the perturbation sensitive zone, can be expressed as:

7 $$ \sigma_{SB} = (\sigma_{s}^{1} + \sigma_{s}^{2} + C^{\prime}\cot \varphi^{\prime})\left( {R_{1} /R_{0} } \right)^{{\frac{{2\sin \varphi^{\prime}}}{{1 - \sin \varphi^{\prime}}}}} - C^{\prime}\cot \varphi^{\prime} $$

Then the unknown parameters $\varphi^{\prime}$ and $C^{\prime}$ are discussed and analysed. The use of bolt support and grouting in the anchorage zone will both improve the mechanical parameters of the fractured surrounding rock. However, because grouting technology is affected by the development of cracks in the surrounding rock, it is only a local regional secondary support measure that is adopted when the cracks are developed to a higher degree. Therefore, the change in the mechanical parameters of the surrounding rock provided by this technology is not considered. Bolt support is a conventional support method in roadways, so it is necessary to consider how it improves the mechanical properties of the surrounding rock in the fractured zone. The bolt’s improvement of the mechanical parameters of the fractured rock mass is mainly reflected in the improved cohesion. Hence, it is assumed that the internal friction angle of the surrounding rock in the anchorage zone remains unchanged, which implies $\varphi^{\prime} = \varphi$. According to relevant research previously, the additional cohesion provided by the bolt to the surrounding rock can be calculated by the following formula:

8 $$ C_{m} = \sigma_{t} \pi d^{2} /4\sqrt 3 S_{c} S_{l} \cos (45^{o} - \varphi /2) $$

where $S_{c}$ and $S_{l}$ are the row and line spacing of the bolts, respectively, $\sigma_{t}$ is the yield strength of the bolt, and $d$ is the bolt’s diameter. Subsequently, the cohesion $C^{\prime}$ of the surrounding rock in the anchorage zone in Eq. (7) can be expressed as:

9 $$ C^{\prime} = C_{0} + C_{m} = C_{0} + \sigma_{s} \pi d^{2} /4\sqrt 3 S_{c} S_{l} \cos (45^{o} - \varphi /2) $$

where $C_{0}$ is the residual cohesion of the fractured rock mass.

By combining the above analysis and calculations, that is by substituting Eqs. (9) and (7) into Eq. (4), the strengthened anti-perturbation strength $\sigma_{lB}^{\prime }$ at the outer boundary of the perturbation sensitive zone can be obtained. Then, by comparing the result with Eq. (3), it can be determined whether the supporting strength meets the requirements of Eq. (2).

## Field application

### Project overview

#### Geological and mining conditions

**Figure 7:**
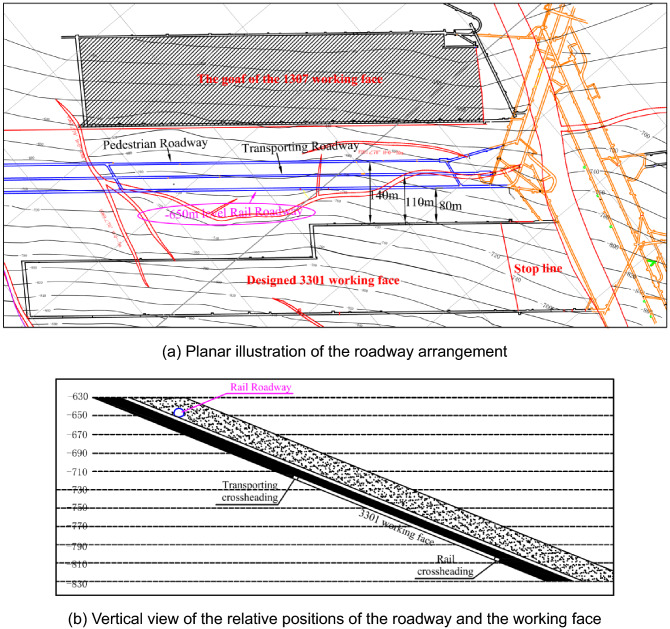
The planar and vertical views of the roadway arrangement.

The Yangcheng Coal Mine is located in the Jining mining area in eastern China. There are three development roadways in the southern mining district at − 650 m depth level: a rail roadway, haulage roadway, and pedestrian roadway. The planar and vertical views of the three roadway arrangements are shown in Fig. 7. The actual cover depth of the three roadways is approximately 800 m, and the original stress level is relatively high. The west side of the three roadways is the goaf of the 1307 working face in the No. 1 mining district. The east side is the designed 3301 working face, as shown in Fig. 7. The closest horizontal distances of the 3301 working face to the rail roadway, transporting roadway and pedestrian roadway are about 80 m, 110 m and 140 m respectively. The main roof’s breakage after mining of 3301 working face induced a cyclic impact loading, which has a greater disturbance effect on the surrounding rock of the three development roadways, especially the rail roadway. To ensure the stability of the surrounding rock during the maintenance of the roadways and reduce the amount of maintenance work, it was decided to optimise the design of the surrounding rock support under the impact of dynamic load. The research took the rail roadway that was closest to the 3301 working face as the engineering object.

The primary mined coal seam of the 3301 working face is seam No. 3. The coal seam’s dip angle is approximately 22°, and the average thickness is 6.5 m. Due to the influence of faults, the design length of the face is 123 m in the early stage and 200 m in the later stage. Comprehensive mechanised caving coal mining method is used for coal extraction. The roof strata above the working face are mainly composed of medium sandstone, mudstone and fine sandstone, and the overall strength of the surrounding rock is medium. The three roadways are all located in the fine sandstone stratum 10.6 m thick above the No. 3 coal seam. The designed cross-section of the rail roadway is approximately circular with a shallow bottom arch. The net height of the roadway is 4200 mm, the net width is 5088 mm and the section radius is 2544 mm. The original ground support system and scheme of the roadway consist of first-stage support utilising a bolt, cable and metal mesh plus a shotcrete layer system, and the secondary reinforcement system using U-type steel support. The pre-tensioned bolt (Ф22 × 2400 mm) was installed with a spacing of 0.7 m by 0.7 m. The 21.6 mm steel strand cable has a length of 7500 mm. The row and line spacing parameters of the bolt arrangement in the rail roadway were 1.6 m by 1.6 m. The metal mesh was welded to a φ5 mm steel bar, and the mesh size was 100 mm by 100 mm. The thickness of the shotcrete was 100 mm. The distance between the adjacent U29 steel supports was 700 mm. It is of note that due to the high level of stress, the initial ground support system controls the long-term instability of the roadway.

#### Static stress and dynamic load

The overall occurrence of the rock strata in the location of the rail roadway is a monoclinic structure that is striking northeast and dipping southeast. The geological structure in this area is complex and there is a large horizontal tectonic stress. According to the test of the static in-situ stress at − 650 m mining level, the maximum principal stress is the horizontal stress is about 23.9 MPa. The k ratio is about 1.31 ~ 1.52. The direction of the maximum principal stress is approximately perpendicular to the strike direction of the rail roadway. According to rock mechanics theory, after the excavation of the roadway, the peak value of concentrated static stress can reach more than twice the in-situ stress level. In addition, the surrounding rock of the rail roadway is prone to large deformation and failure under high concentrated static stress.

The main source of the dynamic load that has a great impact on the rail roadway is the roof breakage after mining of the 3301 working face^25,26^. In the later stage of the mining face, the horizontal distance from the roadway is only about 80 m, and the dynamic impact is obvious. Yangcheng Coal Mine is a typical mine with risk of rock burst, so a real-time microseismic monitoring system has been installed^27–29^. The microseismic monitoring and analysis of the 3304 working face (mining finished) at the same elevation in the same mining district was used to obtain the impacting energy from the overlying basic roof’s periodic break as shown in Table 1. These impacting values can be used as the reference for the impacting loads of the 3301 working face.

**Table 1:** On-site monitoring results of impacting energy caused by basic roof break of the 3301 working face.

Order	Movement stage of main roof	Advancing distances of working face (m)	Source energy (J)	Impacting energy to rail roadway (J)
1	First time break	42	3.38 × 10^5^	4.85 × 10^3^
2	2th break	67	2.15 × 10^5^	2.43 × 10^3^
3	3th break	87	1.98 × 10^5^	2.21 × 10^3^
4	4th break	106	1.86 × 10^5^	2.06 × 10^3^
5	5th break	129	2.11 × 10^5^	2.36 × 10^3^
6	6th break	150	2.02 × 10^5^	2.18 × 10^3^
7	7th break	168	1.88 × 10^5^	1.97 × 10^3^
8	8th break	192	2.20 × 10^5^	2.55 × 10^3^
9	9th break	211	1.90 × 10^5^	2.16 × 10^3^
10	10th break	232	2.10 × 10^5^	2.35 × 10^3^
11	11th break	256	2.18 × 10^5^	2.61 × 10^3^

According to relevant research, the source energy generated after the roof break will gradually attenuate during the propagation within the rock medium, and its attenuation conforms to the law of power exponential decline^30–32^. The distance between the main roof of the 3301 working face and the rail roadway is about 70–80 m. Therefore, the source energy after the roof break of the 3301 working face will be reduced by about two orders of magnitude when propagating to the rail roadway. This is also consistent with the results obtained from on-site monitoring as indicated in Table 1.

### Dynamic parameters test of the surrounding rock

Before designing the support scheme for the rail roadway, the basic rock mechanical parameters and key dynamic parameters of the surrounding rock need to be determined. The authors conducted similar rock creep perturbation experiments on siltstone at the mining level of − 1100 m in Huafeng Coal Mine in eastern China and obtained experimental results^12^. The present study used similar research methods, combined with the process of rock creep perturbation experiments^11,12^, and the dynamic parameters test was also conducted on the fine sandstone of the surrounding rock in the rail roadway.

Our test is a type of creep perturbation experiment under a specific impact load^11,12^. According to the monitoring results of the impacting energy, the average value of the impact energy during ten cycles of roof break was taken as the dynamic impact strength in the experiment, which is 2.288 × 10^3^ J. In the experiment, it was assumed that the gravitational potential energy of weights was completely converted into the impacting energy on the surrounding rock. Through calculation, it was revealed that this impact energy could be achieved by taking a weight of 3 kg and a falling height of 11 cm. In the experiment, the MTS servo rock testing machine was used to measure the basic mechanical parameters of the rock, and the RRTS-II rock creep perturbation experimental system was used to test the rock dynamic parameters. The final experimental results are summarised in Table 2. According to our observations, the laboratory test results of the rock sample’s mechanical parameters are generally higher than the values of the on-site engineering rock mass. Therefore, when applying laboratory test results to the support design of the engineering rock, a weakening ratio needs to be considered. Based on observations in the literature and the in situ geological conditions of Yangcheng Coal Mine and some on-site experiments on the rock mass’s mechanical parameters around the roadway, the weakening ratio was taken as 0.62.

**Table 2:** Test results of the surrounding rock’s mechanical parameters.

Sample	Uniaxial compressive strength (MPa)	Residual strength (MPa)	Cohesion *C* (MPa)	Residual cohesion (MPa)	Internal friction angle (°)	Anti-perturbation strength (MPa)
1^#^	81.2	0.34	23.4	0.073	39	73.7
2^#^	79.4	0.29	24.6	0.065	37	70.3
3^#^	75.5	0.33	20.1	0.072	36	72.3
4^#^	83.1	0.24	23.5	0.07	40	74.5
Average	79.8	0.3	22.9	0.07	38	72.7
Engineering rock mass	49.5	0.3	14.2	0.07	38	45.1

### Design of the support scheme

According to the analysis of the current status of the deep roadway support, the traditional support measures of bolt plus cable and U-type steel support cannot effectively control the large creep and perturbation deformation of the deep roadway in the studied coal mine. Therefore, it was decided to use the composite supporting technology with the CSTS as the main support in the rail roadway. The bolt, metal mesh and concrete spray layer and cables were used as the first step support of the roadway, while the CSTS was used as the secondary step support. Meanwhile, the surrounding rock grouting was used as an alternative technology only when intense development of rock cracks was detected. The supporting technology system described here was used to construct the “bearing circle” structure within the surrounding rock.

#### Design of the first step support

According to the analysis of Eqs. (8) and (9) in Section "Mechanical analysis and determination of support capacity", the improvement provided by the bolts to the mechanical parameters of the surrounding rock mass in the anchorage zone depends on the type of the bolts and their layout parameters. The main type of bolt used in Yangcheng Coal Mine is $\Phi$ 22 × 2400 mm with pre-tension of 5t. The length of the bolt *R*_1_ is the radius of the anchorage zone where *R*_1_ = 2.3 m. The yield strength of the bolt is not less than 335 MPa. For the row and line spacing parameters of the bolt arrangement, if the values are too small, the original integrity of the surrounding rock will be easily damaged; but if they are too large, the bolts cannot effectively improve the mechanical properties of the surrounding rock. According to the previous findings, the supporting density of the bolts is relatively reasonable between 0.6 m and 1.0 m. Therefore, in this study, the designed row and line spacing parameters of the bolt arrangement in the rail roadway were 0.8 m by 0.8 m. According to the test results of the mechanical parameters of the surrounding rock in Table 2, the internal friction angle was 38°, and the residual cohesion was 0.07 MPa. Substituting these parameters into Eqs. (8) and (9) shows that the additional cohesion of *C*_m_ = 0.135 MPa was provided by the bolt to the surrounding rock, and the increased cohesion of the surrounding rock strengthened in the anchorage zone was $C^{\prime}$ = 0.205 MPa.

According to the analysis in Section "Composition of the “bearing circle” supporting technology", the cable can suspend the anchorage zone in a more stable upper stratum, and provide a certain lateral confining pressure for the surrounding rock in the perturbation-sensitive zone. The main type of cable used in Yangcheng Coal Mine is $\Phi$ 21.6 mm. According to the surrounding rock structure, our designed cable length was 8000 mm, considering the local geological condition where the stable main roof is approximately 6-12 m above the excavation. The spacing parameters of the bolt arrangement in the rail roadway were 1.5 m by 1.6 m. The anchoring force of the cable was not less than 250kN. Based on the above parameters, the supporting strength provided by the suspension effect of the cable on the rib of the roadway was $\sigma_{{_{S} }}^{1}$ = 0.104 MPa.

#### Design of the secondary step support

The CSTS structure consists of a seamless steel pipe and internal core-filling concrete. To produce the CSTS structure, an empty steel pipe is bent into the appropriate shape according to the shape of the roadway section; then concrete is poured into the empty steel tube support following the installation of the pipe in the roadway. In order to satisfy the strength needed for the support control of the surrounding rock of the rail roadway, we should have designed the CSTS with Φ194 × 10 mm steel tubing and a concrete strength grade of C40. According to the theoretical algorithm combined with the previous mechanical performance test of the support^21^, this type of CSTS can provide the ultimate bearing capacity of about 2650kN. In the roadway, the CSTS was installed at actual distances of 0.8 m. The radius of the CSTS section at about 2.5 m and of the roadway at *R*_0_ = 2.5 m were about the same. Through calculation, the supporting strength of the CSTS relative to the surrounding rock was determined to be approximately 1.33 MPa, which $\sigma_{{_{S} }}^{2}$ = 1.33 MPa.

#### Verification of support strength

The parameters obtained by the above calculation included: $C^{\prime}$ = 0.205 MPa, $\varphi^{\prime} = \varphi$ = 38°, $\sigma_{{_{S} }}^{1}$ = 0.104 MPa, and $\sigma_{{_{S} }}^{2}$ = 1.33 MPa. In addition, the maximum measured value of the static in-situ stress at − 650 m mining level was about 23.9 MPa, which is *P* = 23.9 MPa. The cohesion of roadway surrounding rock (fine sandstone) under uniaxial compression was 14.2 MPa, which is *C* = 14.2 MPa. The testing result of the surrounding rock’s anti-perturbation strength was 45.1 MPa, which is $\sigma_{lB}$ = 45.1 MPa. By substituting all these parameters into Eq. (3) in Section "Mechanical analysis and determination of support capacity", the concentrated static stress peak at the outer boundary of the perturbation sensitive zone can be obtained as:

10 $$ \sigma_{\theta B} = P(1 + \sin \varphi ) + C\cos \varphi = {49}.{8}0{\text{ MPa}} $$

Bringing all the above parameters into Eqs. (4) and (7) in Section "Mechanical analysis and determination of support capacity", the increased anti-perturbation strength of the surrounding rock at point B after being supported is calculated as:

11 $$ \sigma_{lB}^{\prime } = \frac{1 + \sin \varphi }{{1 - \sin \varphi }}\sigma_{SB} + \sigma_{lB} = \frac{1 + \sin \varphi }{{1 - \sin \varphi }}\left[ {(\sigma_{s}^{1} + \sigma_{s}^{2} + C^{\prime}\cot \varphi^{\prime})\left( {R_{1} /R_{0} } \right)^{{\frac{{2\sin \varphi^{\prime}}}{{1 - \sin \varphi^{\prime}}}}} - C^{\prime}\cot \varphi^{\prime}} \right] + \sigma_{lB} = {54}.{\text{44 MPa}} $$

The relationship between these two parameters is $\sigma_{lB}^{\prime } > \sigma_{\theta B}$, which satisfies the mechanical judging condition required by Eq. (2). This shows that the constructed structure of the “bearing circle” provided sufficient lateral confining pressure for the internal perturbation-sensitive zone. This would make the high concentrated static stress in this zone decreasing out the range of limit strength neighborhood of the surrounding rock, and finally cause the perturbation sensitive zone to disappear. The surrounding rock around the roadway can resist the impact of a cyclic perturbation load of 2.288 × 10^3^ J. Supplementing the roadway with a concrete spraying layer, surrounding rock grouting, and other auxiliary strengthening support measures further improves the bearing performance of the surrounding rock. Thus, the deep roadway can maintain long-term stability under the dual action of high static stress and cyclic external impact load.

#### Support scheme determination of the rail roadway

##### First step support technology

Bolt: $\Phi$ 22 × 2400 mm with high prestress and strength. The bolt spacing in rail roadway was 0.8 m by 0.8 m. The bolt was anchored at full length.

Cable: $\Phi$ 21.6 mm steel strand cable with length of 8000 mm. The row and line spacing parameters of the bolt arrangement in the rail roadway were 1.5 m by 1.6 m. Four cables were installed in every supporting section.

Metal mesh and concrete spray layer: the metal mesh was a square grid, welded by φ5mm steel bars, and the grid size was 100 mm × 100 mm. The thickness of the concrete spray layer was 50 mm, and the concrete strength grade was C20.

##### Secondary step support technology

CSTS: composed of Φ194 × 10 mm steel tube and a concrete strength grade of C40. The selected steel tube’s material was 20# low-carbon steel, which had a yield strength of 245 MPa and a tensile strength of 410 MPa. The distance between two adjacent rows of CSTS was 0.8 m. A type of connecting rod was used to connect the two adjacent CSTSs. The designed shape of the CSTS cross-section was approximately circular with a shallow bottom arch, consistent with the cross-section of the roadway. A 200 mm deformational space was reserved between the roadway-surrounding rock and the CSTS to meet the deformation and stress release requirements of the surrounding rock. The deformation space was filled with flexible blocks.

**Figure 8:**
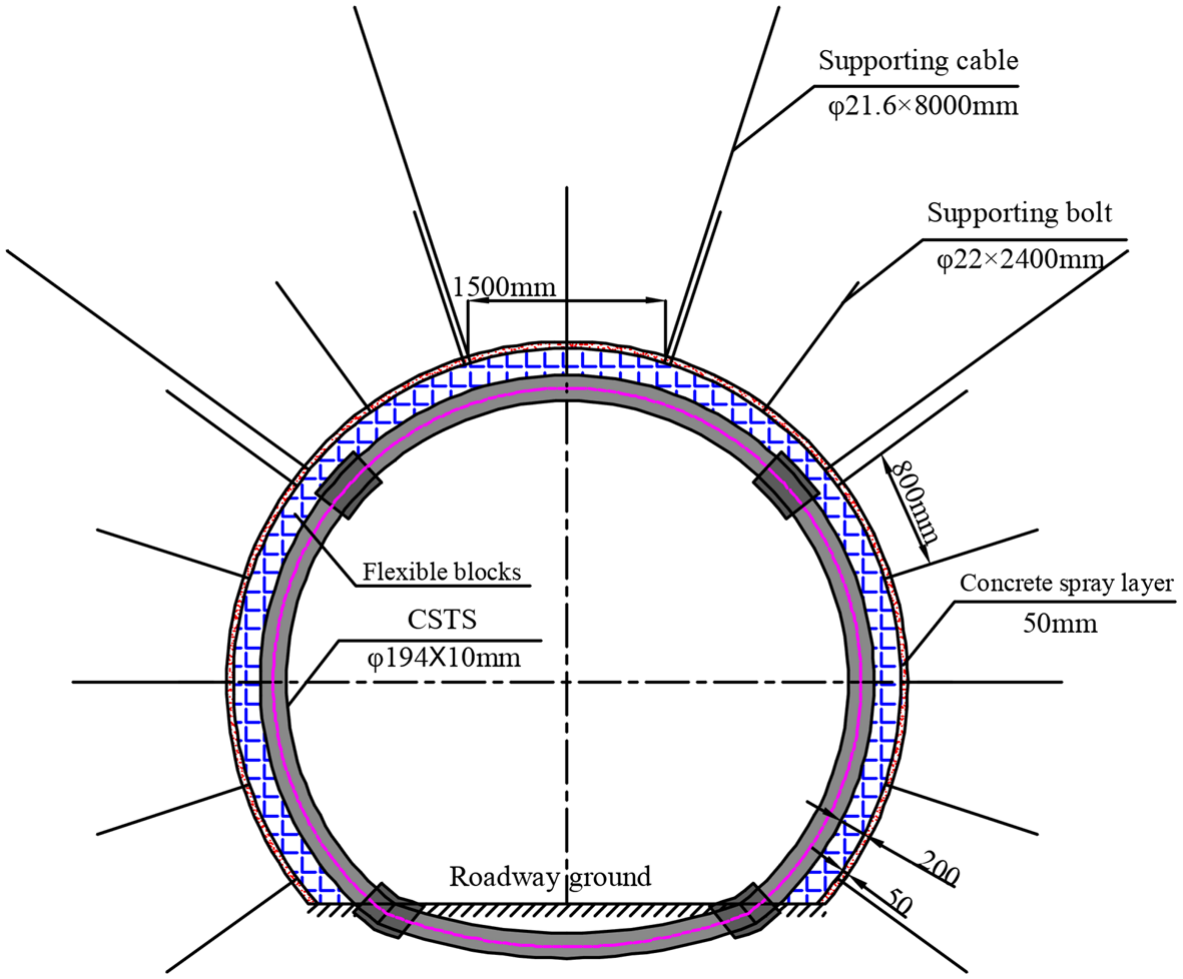
Overall supporting structure in the roadway.

Surrounding rock grouting technology: the slurry material was ordinary single-liquid cement slurry, which was composed of 42.5^#^ ordinary Portland cement and water. The slurry was injected into the surrounding rock cracks with grouting bolts. The design of the overall supporting structure is presented in Fig. 8.

### Engineering application

**Figure 9:**
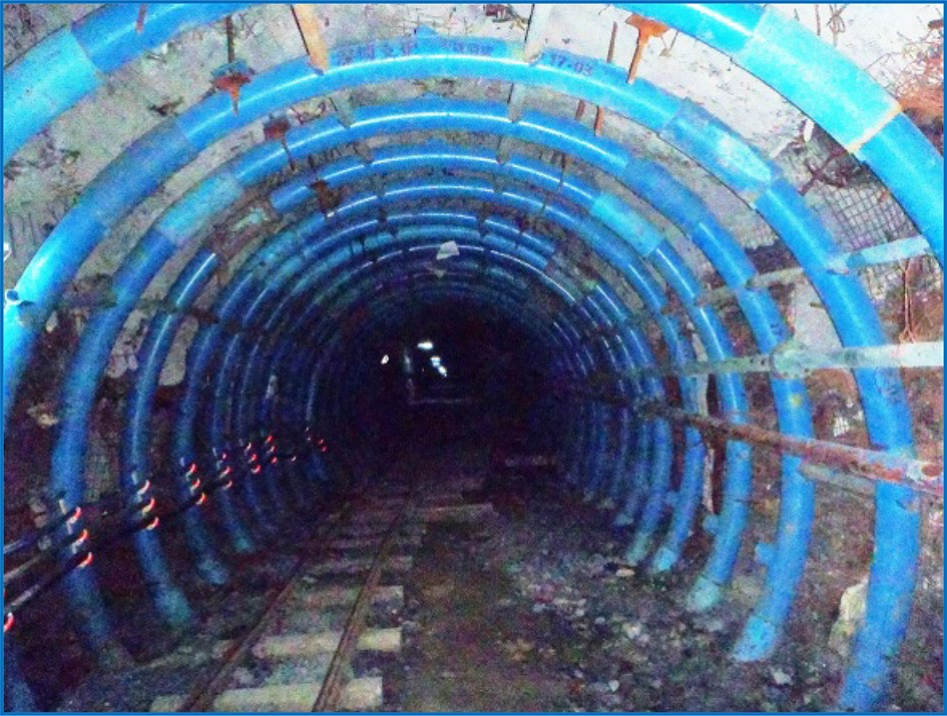
Field application of the CSTS supporting technology.

**Figure 10:**
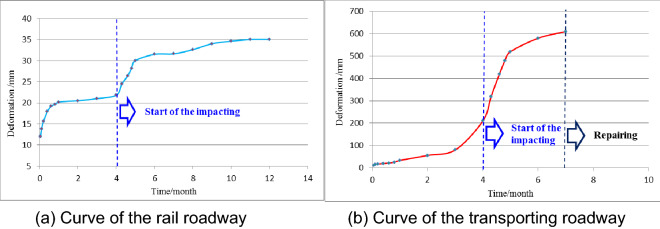
Different deformation curves of the two roadways.

The application of the supporting technology with the CSTS as the main body in the rail roadway is shown in Fig. 9. As a technical comparison, the original supporting technology (bolt, metal mesh and concrete spray layer + cable + U-typed steel support) was used in the transporting roadway. From the beginning of roadway excavation until the completion of 3301 working face mining, the response of the two roadways was obviously different. The comparison curves of the two roadways’ deformations are shown in Fig. 10. For the rail roadway, after the initial bulking deformation, the later impacting perturbation deformation and long-term creep deformation were both well-controlled. One year later, the overall convergence deformation of the roadway was only 35 mm. This shows that the “bearing circle” structure constructed by the new support system has a good bearing effect on the internal surrounding rock and can effectively control the external impact perturbation. The transporting roadway is farther away from the 3301 working face than the rail roadway and is weakly affected by the impact. The amount of bulking deformation of this roadway during excavation is essentially the same as that of the rail roadway. However, due to the lower supporting strength of the U-type steel support, the transporting roadway experienced a continuous large creep perturbation deformation during the mining of 3301 working face. Within 6 months, the overall convergence deformation of the roadway exceeded 600 mm, and a large amount of repair work had to be carried out later.

## Conclusions

This study established a new model of the surrounding rock’s state zone for deep roadways and proposed an improved understanding of the surrounding rock’s deformation mechanism under RFIL. Such deformation is due to the continuous transfer of the perturbation-sensitive zone into the interior surrounding rock under the double effect of greater static stress and the external impact load. The findings were applied to the rail roadway in Yangcheng Coal Mine where the roadway’s long-term creep perturbation large deformation was effectively controlled. To maintain the long-term stability of a deep roadway under the action of RFIL, the key principle is to make the concentrated static stress in the perturbation-sensitive zone redistributing away from the limit strength neighbourhood of the surrounding rock. For this purpose, the most effective measure is to provide the surrounding rock with sufficient lateral confining pressure after excavation.

Then this paper proposed a new dynamic anti-perturbation strength index of rock and established the quantitative design method of support parameters applied in the deep roadway under the action of RFIL. The study proposed the “bearing circle” support system as an innovative supporting principle for a deep roadway affected by RFIL, particular considering the yielding performance of the system under dynamic loading which was limited studies previously.To construct the “bearing circle”, the supporting system was designed with the CSTS as the main body. This is the main technological innovation of this paper.

The in-situ application of the research findings in a deep coal mine in China verified the accuracy and reliability of the proposed theory and design method for support. The proposed method can also be used for different rock types in deep operations, considering the presence of rock discontinuities. Even though the methodology developed in this study proved both theoretically and practically trustworthy, incorporating new technologies such as digital rock technology^33,34^ and machine learning^35,36^ algorithms would further enrich understanding of deep roadways’ surrounding rock and improve design of support systems to prevent mining disasters. In particular, deep learning is a recent development of artificial intelligence that future research can investigate for potential applications.

## Data Availability

The datasets used and/or analysed during the current study are available from the corresponding author on reasonable request.
